# Discovery of a multipotent chaperone, 1-(2,6-Difluorobenzylamino)-3-(1,2,3,4-tetrahydrocarbazol-9-yl)-propan-2-ol with the inhibitory effects on the proliferation of prion, cancer as well as influenza virus

**DOI:** 10.1080/19336896.2020.1714372

**Published:** 2020-01-23

**Authors:** Satoshi Yamashita, Ryo Honda, Mayuko Fukuoka, Tsutomu Kimura, Junji Hosokawa-Muto, Kazuo Kuwata

**Affiliations:** a United Graduate School of Drug Discovery and Medical Information Sciences, Gifu University, Gifu, Japan; b Department of Chemistry, Faculty of Science Division II, Tokyo University of Science, Tokyo, Japan; c First Department of Forsenic Science, National Research Institute of Police Science, Chiba, Japan; d Department of Gene and Development, Graduate School of Medicine, Gifu University, Gifu, Japan

**Keywords:** Chaperone, multipotent, stabilization, free energy, activation free energy, prion, cancer, influenza virus, anti-viral agent, carbazol

## Abstract

We previously discovered three carbazole derivatives, GJP14 (1-piperidinylmethyl-2-(1-oxo-6-methyl-1,2,3,4-tetrahydrocarbazol-9-yl)-ethan-1-ol) with anti-prion activity, GJC29 (benzylamino-3-(1,2,3,4-tetrahydrocarbazol-9-yl)-propan-2-ol) with anti-cancer activity, and THC19 (1-piperidinylmethyl-2-(1,2,3,4-tetrahydrocarnazol-9-yl)-ethan-1-ol) with anti-influenza virus activity. During optimization of GJP14 for the anti-prion activity, we discovered a compound, 1-(2,6-difluorobenzylamino)-3-(1,2,3,4-tetrahydrocarbazol-9-yl)-propan-2-ol, termed 5Y, had the most strong anti-prion activity among a series of newly synthesized derivatives. Intriguingly, we noticed that 5Y had also the most strong anti-colon cancer as well as the anti-influenza virus activities among derivatives. No significant toxicity of 5Y was observed. These results demonstrate that 5Y is a multipotent lead compound with unusually wide spectrum, and may be applicable to therapeutics targeting multiple diseases.

**Abbreviations:** MoPrP: mouse prion protein of amino acid residues of 23-231; PrP^C^: cellular form of prion protein; PrP^Sc^: scrapie form of prion protein

## Introduction

Medical chaperone (MC) hypothesis implies that a compound, which binds to the target conformation of a protein, stabilizes that conformation by reducing its free energy (ΔG) [1]. Although the amount of free energy reduction (ΔΔG) by this mechanism is equivalent to the free energy of binding [2], conformational conversion could also be decelerated by increasing the activation free energy (ΔG^‡^) thereby protecting PrP^C^ from PrP^Sc^ formation. Increase of ΔΔG^‡^ depends on the binding mode, and it is high when the compound binds to the hot spot [3] or the initial converting region (ICR) [4].

In contrast to antibody, MC interacts with the target protein at the local site. Therefore, if the local conformational characteristics are similar between different target proteins, i.e. the local interaction with specific amino acids is predominant compared to that with long-range interaction, MC is able to interact with different proteins. This is a kind of non-specificity, but entirely depending on the local electronic structure of MC. Actually we can apply this inherent non-specificity to design the multipotent MC. Here, we show an excellent example in the carbazole derivatives.

**Figure 1.**
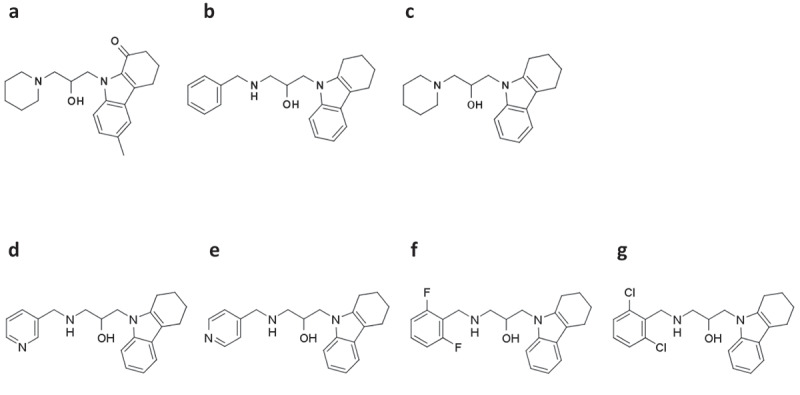
Chemical structures of previously reported anti-prion (GJP14, a), anti-cancer (GJC29, b) or anti-influenza virus (THC19, c) compound, and newly synthesized derivatives, (d) M004, (e) M007, (f) M026, and (g) M027.

Initially, we discovered an anti-prion compound, 1-piperidinylmethyl-2-(1-oxo-6-methyl-1,2,3,4-tetrahydrocarbazol-9-yl)-ethan-1-ol, termed GJP14 [5], whose chemical structure is shown in Figure 1(a), through an *in silico* screen using ICM [6] based on the three dimensional structure of the cellular form of prion protein [7,8]. On the other hand, we independently discovered an anti-cancer compound, benzylamino-3-(1,2,3,4-tetrahydrocarbazol-9-yl)-propan-2-ol, termed GJC29 [9], shown in Figure 1(b), by the similar *in silico* screen using Autodock [10] based on the p53 structure [11]. Eventually, we also independently found a compound, 1-piperidinylmethyl-2-(1,2,3,4-tetrahydrocarbazol-9-yl)-ethan-1-ol, termed THC19 [12], shown in Figure 1(c) with an anti-influenza virus activity, where we found that THC19 might act on the PA [12].

As shown in Figure 1(a–c), chemical structures of GJP14, GJC29 and THC19 commonly include a carbazole moiety, and overall structures are quite similar. We initially optimized the chemical structure of GJP14 in terms of its anti-prion activity [13], and found several newly synthesized compounds with the remarkable anti-prion activities. Among synthesized derivatives, we found the strongest anti-prion compound, 5Y. However, besides its anti-prion activity, here, we have discovered that 5Y has the unusually wide spectrum over cancer as well as influenza virus. Moreover, we discuss the essential role of the local interaction between ligand and target protein, which may cause the non-specificity, and its application.

## Results

**Figure 2.**
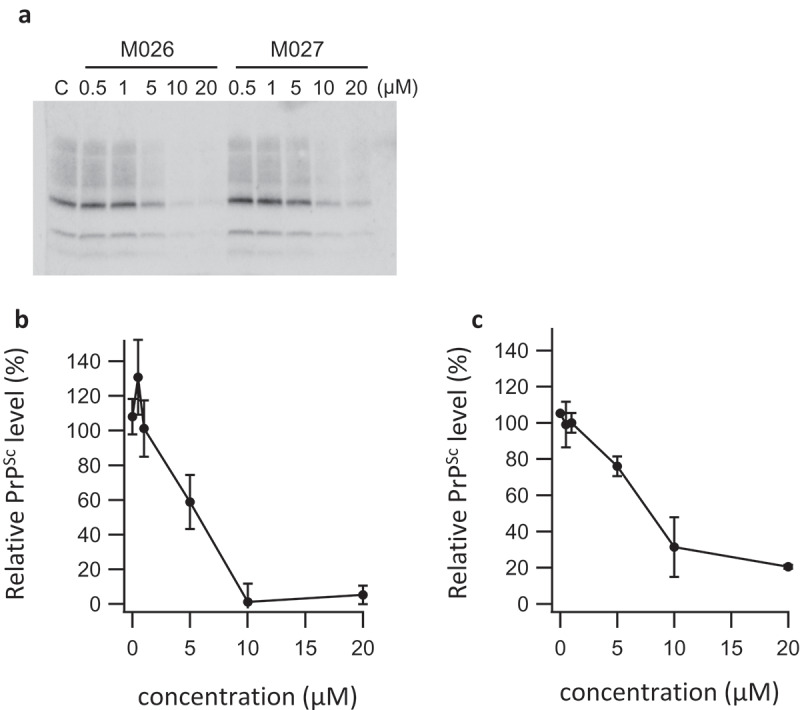
Anti-prion activities. (a) Western blot of prion in GT-FK cells after treatment with M026 and M027. We used 0.5% DMSO as a control. (b) Relative PrP^res^ level as a function of M026 concentration. IC_50_ was calculated to be 4.67 ± 4.93μM. Error bars represent standard deviation of three independent trials. (c) Relative PrP^res^ level as a function of M027 concentration. IC_50_ was calculated to be 7.23 ± 1.65 μM. Error bars represent standard deviation of three independent trials. For curve-fitting, we used Igor Pro 6.12.

Compounds M004, M007, M026, M027 shown in Figure 1(d–g), respectively, which were derivatives GJP14, were tested in GT-FK cells. As shown in Figure 2(a,b), M026, 1-(2,6-difluorobenzylamino)-3-(1,2,3,4-tetrahydrocarbazol-9-yl)-propan-2-ol termed 5Y had the strongest anti-prion activity with IC_50_ of 4.7 μM. Although this IC_50_ value is less than that of GN8, 1.4 μM [3], it is the smallest among the synthesized derivatives. These results are essentially consistent with those of our previous report [13].

An activity of anti-prion compounds sometimes depends on the cell strain. To ensure the strain independent activity of 5Y [3,5], here we examined using the ScN2a-3-Ch [14], which produces larger amount of PrP^Sc^ and thus more robust than GT-FK. Although IC_50_ value was 26.8 μM as shown in Table 1, 5Y exhibited the anti-prion activity on ScN2a-3-Ch cells [14].

**Table 1.** Antiprion activities of derivatives (IC_50_ (μM)).

	M004	M007	M026	M027
GT-FK	13.8	-	4.67	7.23
ScN2a-3-Ch	-	-	26.8	-

**Figure 3.**
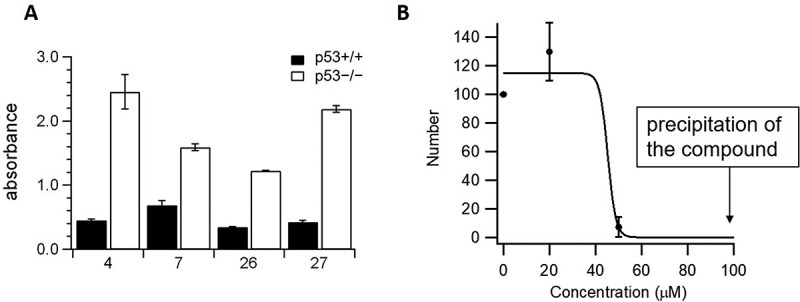
(a) Anti-cancer activities of M004, M007, M026 and M027 at the concentration of 100 μM. HCT116 p53^+/+^ (black) and HCT116 p53^−/-^ (white) cell lines were used. The absorbance of control non-treated cells was approximately 2 (data not shown). (b) Anti-influenza virus activities of 5Y. Other derivatives did not exhibit the anti-influenza activity. Number of colonies was plotted as a function of concentration of 5Y. IC_50_ was calculated to be 45.3 ± 2.56 μM. Error bars represent standard deviation of three independent trials. For curve-fitting, we used Igor Pro 6.12.

Synthesized derivatives were also added into the medium with colon cancer cell, HCT116 p53^+/+^ (wild type) cells [9] to check their anti-cancer activity. Some compounds inhibited the proliferation of cancer cell with higher efficiency than the original compound, GJC29 (data not shown). We also examined their anti-cancer activities for HCT116 p53^−/-^ (null type) [15], and they showed much higher effect in p53^+/+^ cells than in p53^−/-^ cells, as shown in Figure 3(a). It should be also emphasised that most of p53−/− cells survived after the treatment of compounds (50 − 100%) at the concentration of 100 µM, ruling out the possibility of an off-target toxicity. All these results indicate that M026 (5Y) has the remarkable strong anti-cancer activity.

We also examined anti-influenza virus activity of 5Y in MDCK cells inoculated with influenza virus. Most compounds did not show any effect. Although 5Y has less solubility in water, it certainly has an activity with IC_50_ of ~ 40 µM, as shown in Figure 3(b).

## Discussion

Carbazole derivatives have a variety of biological activities, such as an active antagonist for neuropeptide receptor [16] or inhibitors of Hsp90 [17]. Interestingly, Boeckler et al. found a phikan059, a carbazole derivative binds to p53 and is expected to rescue a destabilized mutant of p53 [18]. In our experience, carbazole derivatives have been frequently selected during the *in silico* screen process. This tendency is also considered to be inherently associated with the *in silico* screen strategy using carbazole.

Although there is no similarity in the global three dimensional structures between PrP^C^, p53 or PA, their local structures around binding sites include some resemblance in terms of the interaction with the small compound including carbazole. Carbazole moieties can form π-π[19] or CH-π interactions with aromatic side chains in amino acids, such as tryptophan in p53 [9]. Using Autodock ver. 3.05 [20], we confirmed the binding site of 5Y was the same as GJC29 [9], and its binding mode was quite close to Figure 2(a) in ref. 9 (data not shown). Antibodies can cover large areas in protein surfaces, but small compounds can cover only limited spaces, thus some common moieties among ligands which can effectively bind to the common amino acid side chains might be essential and the other part may exert some specific actions, which depend on the details of the specific electron environments around the binding sites of target protein. Thus, the ligand with the strong binding affinity among its derivatives may also bind to other target, producing non-specificity. However, on the other hand, this kind of non-specificity may produce the wide spectrum over the multiple target diseases, if its chemical structure could be carefully designed.

5Y may interact with the activated conformation (scarcely populated high energy state [3]) of a prion protein (PrP*[21]) at the hot spot or ICR, while p53 amyloid formation may lead to the cancer pathogenesis [22]. Thus, 5Y may interact with the hot spot [9] of the amyloid formation in p53 and prevent the loss of function. In contrast, inhibitory mechanism on the influenza virus proliferation is still unknown, but we may expect that 5Y may interact with a protein via similar mechanism (CH-π or ππ interactions) in influenza virus [23] inhibiting its normal enzymatic function, which would be exerted in the activated state.

GJC29 (Figure 1(b)) was tested *in vivo* for its anti-cancer effect using the nude mice, and the sizes of the implanted colon cancer mass remarkably decreased upon the injection of GJC29 [9]. Thus, *in vivo* studies of the compound 5Y over the anti-cancer, anti-prion and anti-influenza effects are entirely feasible. *In vivo* examination for the anti-prion activity [3], anti-influenza virus activity [24] and also for the anti-cancer activity as well as *in vivo* toxicity studies for compound 5Y would be conducted in near future.

## Methods and materials

Organic syntheses were performed as previously described [13]. Anti-prion assay was performed as previously described [5]. We used an immortalized neuronal mouse cell line (GT-FK cells) that was persistently infected with mouse-adapted GSS agent (Fukuoka-1 strain) [25] and N2a subclone infected with the Chandler strain (ScN2a-3-Ch cells) [14]. Cells were cultured in Dulbecco’s Modified Eagle Medium (Life Technologies) supplemented with 10% FBS (Life Technologies), 50 units/ml of penicillin and 50 μg/ml Streptomycin (Life Technologies). Compounds were dissolved in dimethyl sulphoxide (DMSO) at a stock concentration of 10 mM. Approximately, 3 × 10^5^ cells were seeded into 6-well plate. Three days after the addition of the compound, cells were lysed with 150 μL of 1× Triton-DOC lysis buffer (150 mM NaCl, 0.5% Triton X-100, 0.5% sodium deoxycholate, and 50 mM Tris–HCl [pH 7.5]). After 1 min of centrifugation at 11,200 × g, the total protein concentration of the supernatant was determined by the BCA protein assay (Thermo Fisher Scientific, Rockford, IL) and adjusted with the lysis buffer to 1 mg/mL. The samples were digested with with 20 μg of proteinase K per mL and centrifuged at 22,000 × g for 45 min at 4°C. The pellets were resuspended in 1× Lammli sample buffer and then loaded onto a polyacrylamide gel after boiling. Then, Western blotting for PrP^Sc^ was performed. To detect PrP^Sc^, PrP M-20 antibody (Santa Cruz Biotechnology, Santa Cruz, CA) was used as a first antibody and fluorescently-labelled monoclonal antibody as a second antibody. These signals were visualized by Super-Signal (Pierce Biotechnology, Rockford, IL). The density of PrP^Sc^ was measured and compared those of test compounds with that of the control.

The anti-cancer assay was performed as described [9]. The human colon cancer-derived cell line, HCT116 was purchased from ATCC. The isogenic HCT116 p53^+/+^ cells and HCT116 p53^−/-^ cells were kindly provided by Prof. Bert Vogelstein (The Johns Hopkins University, Baltimore, MD, USA). Cells were cultured in DMEM supplemented with 10% FBS, 50 units/ml of penicillin and 50 μg/ml Streptomycin (Life Technologies). Approximately, 0.5 × 10 [4] cells were seeded into 96-well plate, and grown for 2 days. Twenty-four hours after the addition of compounds, cell viability was measured by WST-8 assay (Cell Countin Kit-8, Dojindo, Japan).

Madin-Darby canine kidney cells (MDCK cells) were kindly provided by Professor Hideto Fukushi, United Graduated School of Veterinary Sciences, Gifu University. MDCK cells were maintained in minimal essential medium α (Wako) supplemented with 10% FBS, 50 U/ml penicillin and 50 μg/ml streptomycin (Life Technologies). The influenza A virus strain A/WSN/33 H1N1 was provided by Professor Yoshihiro Kawaoka, the Institute of Medical Science, the University of Tokyo. MDCK cells (1 × 10^6^cells/well) were seeded in a 6-well plate and pre-cultured for 24 h. A monolayer of MDCK cells was washed with serum-free MEMα, and influenza virus were adsorbed onto the cells for 1 h. The cells were washed and overlaid with MEM 2x (Life Technologies) supplemented with 1 mg/ml BSA, 6 μg/ml trypsin, Non-Essential Amino Acid (Life Technologies), 1% low melting temperature agarose, INA-Agar BA-30 (Funakoshi, Japan), and 0–100 µM compound. After 3 days incubation, the cells were stained with crystal violet, and the number of plaques were manually counted [23].
